# *Mycoplasma genitalium* in the Far North Queensland backpacker population: An observational study of prevalence and azithromycin resistance

**DOI:** 10.1371/journal.pone.0202428

**Published:** 2018-08-28

**Authors:** Thomas Trevis, Marianne Gossé, Nicola Santarossa, Sepehr Tabrizi, Darren Russell, William John McBride

**Affiliations:** 1 James Cook University Clinical School, Cairns Hospital, Cairns, Queensland, Australia; 2 Norwegian University of Science and Technology, Faculty of Medicine, Trondheim, Norway; 3 Department of Obstetrics and Gynaecology, University of Melbourne, Victoria, Australia; 4 Cairns Sexual Health Service, Cairns North, Queensland, Australia; Academia Sinica, TAIWAN

## Abstract

**Background:**

*Mycoplasma genitalium* is a sexually transmitted infection (STI), and a common cause of non-gonococcal urethritis (NGU). There is concern regarding the rise in prevalence of *M*. *genitalium* and rates of resistance to macrolide antibiotics. International backpackers represent a unique population that may be at an increased risk of STIs. The purpose of this study was to determine the prevalence of *M*. *genitalium* and antibiotic resistance in international backpackers.

**Methods:**

First void urine samples were obtained utilising opportunistic sampling from 294 non-treatment-seeking international backpackers at a variety of hostels in Cairns, Queensland Australia. Participants also answered a fixed-answer survey regarding sociodemographic characteristics and sexual risk behaviours. Samples were tested for *M*. *genitalium*, *Chlamydia trachomatis* and *Neisseria gonorrhoeae* using polymerase chain reaction (PCR). Samples positive for *M*. *genitalium* were investigated for macrolide resistance-associated mutations in the 23S rRNA genome at positions A2058G, A2058C, A2058T, A2059G and A2059C (*Escherichia coli* numbering).

**Results:**

Of the 294 samples, 23 failed the internal control. The prevalence of *M*. *genitalium* was 1.8% (5/271, 95% confidence interval [CI] ± 1.58), *C*. *trachomatis* was 4.1% (11/271, 95% CI ± 2.36) and *N*. *gonorrhoeae* was not detected. Macrolide resistance-associated mutations were identified in 40% (2/5) of *M*. *genitalium*-positive samples. *M*. *genitalium* infection was associated with reporting symptoms (odds ratio [OR] 14.36, 95% CI 2.17–94.94, p < 0.05).

**Conclusions:**

*M*. *genitalium* and *C*. *trachomatis* are relatively common amongst non-treatment seeking international backpackers, but may not differ from Australian population prevalence. This article provides evidence to further support the increased utilisation of *M*. *genitalium* PCR in the diagnosis of NGU, and for macrolide resistance testing for all identified *M*. *genitalium* infections.

## Introduction

*Mycoplasma genitalium* is a sexually transmitted infection (STI) and an established cause of non-gonococcal urethritis (NGU), being isolated as the most common pathogen in 6–50% of people presenting with NGU [1]. Population prevalence of *M*. *genitalium* is estimated in a national Danish study, in a range of 1.1–3.3%; with this same study also suggesting that the prevalence of *M*. *genitalium* may be increasing [2]. In Australia, the prevalence of *M*. *genitalium* rages from 2.1–13% depending on the population tested [3–6]. The majority of people infected with *M*. *genitalium* are likely to be asymptomatic, with 52% asymptomatic in one cohort study [7,8].

### Clinical symptoms

Up to 6% of male urogenital complaints may be caused by *M*. *genitalium*, which may include: balanoposthitis, urethral discharge and/or dysuria [9–15]. *M*. *genitalium* may also be associated with chronic prostatitis and epididymitis [11–16]. In females, *M*. *genitalium* is associated with cervicitis, pelvic inflammatory disease, urethritis and infertility [17–19]. *M*. *genitalium* has also been associated with preterm birth and spontaneous abortion [19].

### Treatment of *M*. *genitalium*

In Australia, the mainstay treatment of NGU is single dose 1 gram azithromycin or a 7-day course of doxycycline 100 mg twice daily [20]. One review of recent literature concluded that single-dose azithromycin can induce macrolide resistance and is ineffective if macrolide resistance is present [7]. Although more efficacious than doxycycline (failure rates of up to 83%), azithromycin therapy only resulted in cure of *M*. *genitalium* in 40–88% of cases; however, this depended on population resistance levels [7]. Prolonged courses of azithromycin, although slightly more efficacious and less likely to induce macrolide resistance, also remained ineffective if macrolide resistance is already present [7]. In cases of resistance, moxifloxacin 400 mg once daily for 10–14 days remained effective in > 88% of cases, however concerns exist regarding emerging fluoroquinolone resistance in *M*. *genitalium* [7].

### Macrolide resistance

Resistance to macrolide class antibiotics has become an increasing concern both within recent literature and in clinical practice, with resistance in *M*. *genitalium* reported as high as 58.0% in one study investigating attendees at a Canadian sexual health clinic [8]. The first study demonstrating resistance of *M*. *genitalium* to macrolide antibiotics, namely azithromycin, was published in 2006 [21]. In 2008, three mutations at the 2058 and 2059 (A2058G, A2059G and A2058C, *Escherichia coli* numbering) positions in the region V of the 23S rRNA gene were identified to confer clinical macrolide resistance; findings that were consistent with similar research in other *Mollicutes* [22,23]. Two additional mutations (A2059C and A2058T) were identified using high resolution melt analysis in 2012 [24]. Macrolide resistance-associated mutations have been shown to correlate with microbiological and clinical treatment failure (p = 0.013 and p = 0.024, respectively) [25].

In Australian studies, treatment failure with azithromycin has increased from 16% in a 2005–2007 cohort, to 31% in a 2007–2009 cohort of the same patient population [24,26]. In a recent prospective cohort study, also conducted in Australia, treatment failure with single-dose azithromycin was seen in 39% of participants [27]. Macrolide resistance-associated mutations were detected in 36% of patients, of which 87% failed single-dose azithromycin treatment [27]. These studies suggest that in Australia, resistance-associated mutations in *M*. *genitalium* are becoming increasingly prevalent. Alarmingly, a recent study in Greenland detected macrolide resistance-associated mutations in 100% of *M*. *genitalium* positive samples [28].

### *M*. *genitalium* and international travellers

*M*. *genitalium* prevalence and antibiotic resistance has not been described in any travelling population. International backpackers are defined as any international traveller staying at least one night in a hostel-style accommodation, and represents an under-studied population in Australia [29]. The prevalence of STI amongst international travellers ranges between 1.6–27.1% for *C*. *trachomatis*, 0–36.7% for *N*. *gonorrhoeae*, 1.3–24.5% for *Treponema pallidum* (syphilis), 0.07–1.3% for *Trichomonas vaginalis*, 1.3–24.5% for herpes simplex virus (HSV), 6.4–9.6% for human papillomavirus (HPV) and 0–4.1% for HIV depending on the study design and specific population sampled [30–39].

Sexually transmissible infections and high-risk sexual behaviour, such as inconsistent condom use and casual sex, alcohol use and engaging in illicit drug use, are reasonably common amongst international travellers in Australia[30–35,40]. During travel, 48–94% of backpackers reported engaging in new sexual partnerships and 41–80% reported inconsistent condom use with new sexual partnerships whilst in Australia [30–34,40]. For this reason, is it believed that backpackers may represent a group at high risk of being infected with *M*. *genitalium*.

The aims of this study are to describe the point prevalence of *M*. *genitalium* and rates of macrolide resistance-associated mutations within the Far North Queensland backpacker population. Additional primary aims include establishing correlations between *M*. *genitalium* positivity and sexual risk behaviour, such as frequency of sexual partners, condom use and travel history.

## Methods

### Participants

This cross-sectional population-based study was undertaken in Cairns, Queensland Australia between the months of May and June of 2016. During this period, first void urine (FVU) samples and survey data were collected from international backpackers staying in hostel-style accommodation using opportunistic sampling. Permission was sought from the management of these hostels to recruit participants on their premises, provide sexual health information and distribute condoms and water-based lubricants. Hostels in three suburbs of Cairns were contacted, including: sixteen hostels in Cairns City (six agreed to participate) three hostels in Cairns North (all agreed) and one hostel in Parramatta Park (agreed). These hostels were visited in the afternoons and evenings in an attempt to maximise the number of people engaged, as travellers tended to return to hostels from tourist activities in the late afternoon. The research team returned to locations repeatedly, often leaving one or two weeks between visits, until the target sample size was reached. Subjects were only allowed to participate once, however those who requested an additional STI screen were welcome to have one; these additional results did not form part of our dataset. We anticipated a conservative prevalence of 3% in this population, therefore 279 participants would be required to obtain a 95% CI of ± 2%; this figure represents our minimum sample size.

International backpackers aged 18 years or older who have stayed, or are planning to stay, at least 1 night in a backpacker hostel were included in this study. Participants were required to have the capacity to provide informed consent, including sufficient English proficiency to fully understand the consent form. Exclusion criteria prevented the enrolment of those under the age of 18 years and Australian citizens. Participants were approached at random and were provided both written and verbal information about the project. Confectionaries were offered to those who participated, with condoms, personal lubricant, and information about STIs and accessing healthcare in Cairns were freely distributed.

### Questionnaire

Written informed consent was obtained from all participants prior to commencing the online survey using the program Google Forms™ (Google Incorporated). Participant provided information included: sociodemographic characteristics, arrival with or without a partner, whether they had stayed at least one night in a backpacker hostel, and their total time thus far in, and total expected stay in, both Australia and Cairns. Included in this survey were a range of questions regarding sexual behaviour, including: the number of sexual partners in the 12 months prior to leaving their country of origin, the number of sexual partners and condom use with these partners whilst in Australia and in Cairns specifically. Travel to a country prior to visiting Australia, the number of sexual partners and condom use in this country were also recorded. Finally, participants provided responses for whether they currently experienced symptoms typical of an STI, whether they had been diagnosed with an STI in the past and whether they had received antibiotic therapy in the last three months S1 File. Participant’s responses were de-identified prior to statistical analysis and re-identified in the event of a positive result. Data were stored in Google Drive™ (Google Incorporated) which requires 2-factor authentication to retrieve.

### Specimen collection and analysis

Participants were provided with verbal instruction and asked to supply a FVU sample, all of which were self-collected by participants. Urine specimens were transferred to 1.5 mL micro centrifuge tubes within 3 hours of collection and immediately frozen at –20 degrees Celsius in a freezer with 24-hour temperature monitoring. Following each 4-week period of data collection, samples were sent in a thermal box with a frozen gel pack via overnight courier to The Women’s Hospital in Melbourne, Victoria to undergo molecular testing in two batches. Detection of *M*. *genitalium* and macrolide resistance was undertaken simultaneously, utilising the SpeeDx PlexPCR™ *M*. *genitalium ResistancePlus*™ assay (SpeeDx Pty. Ltd., Australia) and Roche LightCycler® 480 instruments (Roche Products Pty. Ltd., Australia), following the manufacturer’s instructions for both. This assay determines the presence of *M*. *genitalium* by detection of the MgPa gene. This assay simultaneously identifies the presence of 5 mutations at positions A2058G, A2059G, A2058C, A2059C and A2058T (*E*. *coli* numbering) in the 23S rRNA genome of *M*. *genitalium* associated with resistance to macrolide-based antibiotics. Sensitivity for the detection of *M*. *genitalium* using this assay is 99.1% and specificity is 98.5% when compared to reference methods (qPCR targeting the 16S rRNA gene). Sensitivity for detection of macrolide resistance associated mutations is 97.4% and specificity is 100% when compared to reference methods (high resolution melt analysis) [41].

Efficacy of the DNA extraction was assessed and the presence of PCR inhibitors was identified by amplifying a 260bp region of the human beta-globin gene, using methods described in prior literature [42,43]. Detection of *C*. *trachomatis* targeting the ompA gene and *N*. *gonorrhoeae* targeting the OPA gene was conducted using published in house-assays [44,45]. Samples testing low-positive for the presence of *N*. *gonorrhoeae* were confirmed by the detection of the PorA pseudogene using methods previously described [46].

### Follow up

Participants with positive test results were contacted with their result and resistance status to best facilitate appropriate care. This was completed by following a semi-scripted ethics-approved re-contact protocol, first by phone (if provided) and then by email.

### Data analysis

Specimen data were also entered into Microsoft Excel™ (Microsoft Corporation) and were linked to matched survey data. Data were coded within Excel and analysed by IBM Statistical Package for the Social Sciences version 23™ (International Business Machines Corporation). Proportions with 95% CI were calculated for *M*. *genitalium* and *C*. *trachomatis* prevalence. Pearson’s chi-squared test for independence (plus Yates’ correction for continuity) and Fisher’s exact test were used to compare categorical variables. Spearman’s ρ correlation coefficient (*r*) was used to compare continuous variables to dichotomous variables (sex, STI positivity, arrival with sexual partner etc.). Differences between male and female correlation coefficients were performed on all statistically significant correlations, where the critical value of z at p = 0.05 level was ≤ -1.96 or ≥ 1.96, with observed z values (z_obs_) outside the critical value of z considered significant.

### Ethics

Ethics approval was granted by the James Cook University Human Research Ethics Committee April 26^th^ 2016. Ethics approval number H6483.

## Results

Data were collected from ten locations over twenty-nine separate occasions during the two-month data collection period. Six participants were removed from the dataset due to selecting Australia as their country of origin on the survey. In total, we recruited 157 males (53.0%) and 139 females (47.0%), resulting in a total of 296 completed surveys and 294 FVU samples (two participants were unable to produce urine samples). Of the 294 FVU samples, 23 failed the internal control due to inhibitors in the urine, resulting in 271 valid urine sample results, all with paired survey data.

### Participant demographics

The median age of all participants was 23.4 years (interquartile range [IQR] 21.4–26.0). For males, the median age was 23.4 (IQR 21.6–26.2) and for females the median age was 23.1 (IQR 20.9–26.0). Twenty-two-point six percent of all participants were aged 18–20 years, 45.3% were aged 21–24 years and 32.1% were aged ≥ 25 years. The majority of participants originated from European countries (85.5%), with the remainder originating from North America (10.5%), South America (1.4%), Asia (1.4%) and Oceania (0.7%); no participants originated from continental Africa. Of the European counties, 37.8% were from the United Kingdom of Great Britain and Northern Ireland, 18.5% were from Germany, 6.4% were from the Netherlands, 5.7% were from France and 4.4% were from Sweden; 12.9% were from other European countries.

### Prevalence of *M*. *genitalium* and macrolide resistance

Five samples tested positive for *M*. *genitalium* out of the 271 valid urine tests, resulting in a prevalence of 1.8% (5/271, 95% CI ± 1.58). None of these five sample were also positive for *C*. *trachomatis* or *N*. *gonorrhoeae*. Two people testing positive for *M*. *genitalium* reported experiencing symptoms, therefore the asymptomatic prevalence of *M*. *genitalium* in this sample was 1.3% (3/240, 95% CI ± 1.43). Independent variables obtained from the survey were explored for associations with *M*. *genitalium* positivity, the only statistically significant association identified was reporting symptoms (odds ratio [OR] 14.36, 95% CI 2.17–94.94, relative risk [RR] 12.31, 95% CI 2.25–67.37, p < 0.05). In males, three samples were positive, resulting in a prevalence of 2.2% (3/135, 95% CI ± 2.47). In females, two samples were positive, giving a prevalence of 1.5% (2/136, 95% CI ± 2.04). Of the five positive samples, macrolide resistance-associated mutations were identified in two, demonstrating a 40% rate of resistance in our sample population. The sociodemographic and sexual risk behaviours exhibited by those subjects positive for *M*. *genitalium* are outlined in Table 1. Antibiotic use in the past 3 months was reported by 18.9% of participants, with the majority (71.4%) unable to recall what was prescribed.

**Table 1:** Sociodemographic and sexual risk behaviour of participants positive for *M*. *genitalium*.

	Subject 1	Subject 2	Subject 3	Subject 4	Subject 5
Resistance mutation	Present	Absent	Absent	Present	Absent
Age (years)	23	18	25	28	25
Gender	Male	Female	Male	Female	Male
Country of origin	Italy	United Kingdom	Canada	Belgium	United States of America
Arrival with partner	No	No	No	No	No
Time spent in Australia	> 12 weeks	> 12 weeks	4–12 weeks	2–4 weeks	> 12 weeks
Time spent in Cairns	> 12 weeks	4–12 weeks	< 2 weeks	< 2 weeks	4–12 weeks
Number of sexual partners prior to travel^^^	> 10	2	1	2	4
Number of new sexual partners in Australia	> 10	3	2	0	5
Number of new sexual partners in Cairns	> 10	1	0	0	3
Frequency of condom use with new partners in Australia	Sometimes	Never	Never	-	Sometimes
Country visited prior to Australia	Spain	-	New Zealand	-	Indonesia
Number of new partners in country prior to Australia^∞^	> 10	-	2	-	3
Frequency of condom use with new partners prior to Australia	Sometimes	-	Never	-	Always
Past diagnosis of an STI	No	No	No	Yes	Yes
Symptomatic^§^	No	Yes	Yes	No	No
Antibiotics in the preceding 3 months	No	No	No	Uncertain	No

^^^. Approximate number of sexual partners in the 12 months prior to leaving the country of origin.

^∞^. Approximate number of sexual partners in the country visited immediately prior to entering Australia, not including stopovers ≤ 24 hours in length.

^§^. Symptoms including: pain when urinating, pain with sexual intercourse, discharge or other symptoms that would suggest to participants that they may have an STI

### Prevalence of *C*. *trachomatis* and *N*. *gonorrhoeae*

Eleven samples tested positive for *C*. *trachomatis*, resulting in a prevalence of 4.1% (11/271, 95% CI ± 2.36) in our sample population. The number of evaluable asymptomatic subjects was 240, adjusted from 263 for the 23 samples from asymptomatic individuals whose samples exhibited PCR inhibition. Ten participants who tested positive for *C*. *trachomatis* denied symptoms and one was uncertain, resulting in an asymptomatic prevalence of 4.2% (10/240, 95% CI ± 2.54). In males, six samples were positive, resulting in a prevalence of 4.4% (6/135, 95% CI ± 3.46). In females, five samples were positive, resulting in a prevalence of 3.7% (5/136, 95% CI ± 3.17). Independent variables obtained from the survey were explored for associations with *C*. *trachomatis* positive. Those participants arriving in Australia with a sexual partner were more likely to test positive for *C*. *trachomatis* (OR 5.21, 95% CI 1.27–20.34, p < 0.05), however the effect size was small (Cramér’s V [V] = 0.15). There was small but significant correlation between the number of sexual partners engaged with in Cairns and positivity for *C*. *trachomatis* (*r* = .131, p < 0.05), this correlation appeared to be stronger and significant in females (*r* = .208, p < 0.05 vs *r* = .038, not significant); however, the correlation coefficient did not differ significantly between groups (z_obs_ = -1.39). No other statistically significant associations were found between *C*. *trachomatis* positivity and surveyed variables, including symptoms. Nine samples were low-positive for *N*. *gonorrhoeae* on initial testing, these were shown to be equivocal on confirmation testing of the PorA pseudogene sequence, and therefore considered negative.

### Sexual risk behaviour

Table 2 summarises the sexual behaviour of participants, categorised by gender. Twenty-one percent of participants arriving in Australia without a sexual partner engaged in sex with one partner, and 61.0% engaged in sex with 2 or more partners, whilst in Australia. Twenty-point six percent engaged in sex with one sexual partner whilst in Cairns and 24.6% engaged with 2 or more partners in Cairns. Of those engaging in sex with new partners whilst in Australia, 24.5% always used condoms, 38.6% sometimes used condoms, 10.5% used condoms infrequently, and 26.4% never used condoms.

**Table 2:** Reported sexual behaviour by gender.

Sexual Behaviour	Males		Females		Total	
	*n*/total	%	*n*/total	%	*n*/total	%
Arrival with partner						
Yes	13/157	8.3	11/139	7.9	24/296	8.1
No	144/157	91.7	128/139	92.1	272/296	91.9
Number of sexual partners prior to travel^^^						
0	5/157	3.2	6/139	4.3	11/296	3.7
1	33/157	21.0	40/139	28.8	73/296	24.7
2–5	67/157	42.7	69/139	49.6	136/296	45.9
≥ 6	52/157	33.1	24/139	17.3	76/296	25.7
Number of new sexual partners in Australia^Δ^						
0	31/144	21.5	18/128	14.1	49/272	18.0
1	28/144	19.4	29/128	22.7	57/272	21.0
2–5	47/144	32.6	52/128	40.6	99/272	36.4
≥ 6	38/144	26.4	29/128	22.7	67/272	24.6
Number of new sexual partners in Cairns^Δ^						
0	81/144	56.3	68/128	53.1	149/272	54.8
1	30/144	20.8	26/128	20.3	56/272	20.6
2–5	22/144	15.3	28/128	21.9	50/272	18.4
≥ 6	11/157	7.6	6/128	4.7	17/272	6.3
Condom use with new partners in Australia^‡^						
Always	32/111	28.8	22/109	20.2	54/220	24.5
Sometimes	40/111	36.0	45/109	41.3	85/220	38.6
Infrequently	11/111	9.9	12/109	11.0	23/220	10.5
Never	28/111	25.2	30/109	27.5	58/220	26.4
Number of sexual partners en route to Australia^∞^^†^						
0	30/77	39.0	33/58	56.9	63/135	46.7
1	19/77	24.7	10/58	17.2	29/135	21.5
2–5	20/77	26.0	10/58	17.2	30/135	22.2
≥ 6	8/77	10.4	5/58	8.6	13/135	9.6
Condom use with partners en route to Australia^◊^						
Always	16/46	34.6	8/25	32.0	24/71	33.8
Sometimes	21/46	45.7	12/25	48.0	33/71	46.5
Infrequently	2/46	4.3	-	-	2/71	2.8
Never	7/46	15.2	5/25	20.0	12/71	16.9
Past diagnosis of an STI						
Yes	29/157	18.5	35/139	25.2	64/296	21.6
No	126/157	80.3	102/139	73.4	228/296	77.0
Unsure	2/157	1.3	2/139	1.4	4/296	1.4
Symptomatic^§^						
Yes	3/157	1.9	10/139	7.2	13/296	4.4
No	143/157	91.1	120/139	86.3	263/296	88.9
Unsure	11/157	7.0	9/139	6.5	20/296	6.8

^^^. Approximate number of sexual partners in the 12 months prior to leaving the country of origin.

^∞^. Approximate number of sexual partners in the country visited immediately prior to entering Australia, not including stopovers ≤ 24 hours in length.

^†^. Data expressed as the proportion of participants who visited another country en route to Australia.

^Δ^. New sexual partners are defined as those participants who reported arriving in Australia without a partner who engaged in sexual intercourse whilst in Australia or Cairns.

^‡^. Condom use in those who arrived in Australia without a partner and engaged in sexual intercourse, note that 2 male participants and 1 female participant provided invalid responses.

^◊^. Condom use in those reporting engaging in sexual intercourse in the country visited en route to Australia, note that 1 male participant provided an invalid response.

^§^. Symptoms including: pain when urinating, pain with sexual intercourse, discharge or other symptoms that would suggest to participants that they may have an STI

## Discussion

In this cross-sectional population study of 294 non-treatment-seeking international backpackers in Cairns, Queensland Australia, the prevalence of M. genitalium infection was 1.8%, the prevalence of *C*. *trachomatis* was 4.1% and the prevalence of *N*. *gonorrhoeae* was 0%. Macrolide resistance-associated mutations were identified in 40% of *M*. *genitalium*-positive samples. Despite evidence of high risk sexual behaviour, with 61.0% engaging in sexual activity with multiple partners and 75.5% reporting inconsistent or absent condom use, prevalence of M. genitalium was less than that of other Australian population groups, such as 13% in at-risk youth accessing health services, 2.1% in men who have sex with men in men-only saunas and 2.4% in females attending primary healthcare services [6,47,48]. Only one other study has investigated the prevalence of *M*. *genitalium* and macrolide resistance in the same geographical area, in a cohort of incarcerated men, where the prevalence was 5.71% and macrolide resistance was 25% [3]. High rates of macrolide resistance have also been reported in other centres within the state of Queensland, with one study reporting macrolide-associated resistance mutations in 63.6% of samples collected from 2011–2017 [49]. The rates of macrolide resistance-associated mutations identified in this study are consistent with that of prior Australian research, and support the notion that resistance is increasing in Australian samples, with 16% identified in a 2005–2007 cohort, 19.5% in a 2007–2008 cohort, 43% in a 2008–2011 cohort, 25% in a 2012–2013 cohort to 40% in our 2016 cohort [3,24,26,50].

Our research supports statements in recent literature calling for increased molecular testing for *M*. *genitalium* in primary healthcare environments and recall for test of cure in all patients presenting with *M*. *genitalium*-positive NGU [7]. This is becoming increasingly pertinent given the growing body of evidence supporting the long-term and irreversible negative consequences of *M*. *genitalium* infection in women. Given the accumulative evidence for the role of *M*. *genitalium* in NGU and increasing prevalence of macrolide resistance, our study supports the call to update empirical treatment guidelines for NGU. This is relevant given the overall lack of access to, and utilisation of, *M*. *genitalium* PCR testing resulting in a considerable reliance on empirical guidelines in the treatment of NGU. Following the advent of commercially available assays that simultaneously detect *M*. *genitalium* and macrolide resistance-associated mutations, increased application of molecular techniques in the diagnosis of NGU and in asymptomatic screening programs have the potential to accurately guide appropriate antibiotic therapy, reduce transmission of resistant strains and prevent long-term consequences of asymptomatic infection [24,41]. In line with recent research, the guidelines for management of PCR-proven *M*. *genitalium* infection recommenced by the Australian STI Management Guidelines have changed. Antimicrobial recommendations for *M*. *genitalium* infection known or suspected to be susceptible to macrolides includes doxycycline 100 mg twice daily for seven days followed by either azithromycin 1 gram as a single dose or azithromycin 1 gram followed by 500 mg for three days. *M*. *genitalium* infection known or suspected to be resistant to macrolides is recommended to be managed with doxycycline 100 mg for seven days followed by moxifloxacin 400 mg for seven days [51]. Despite this further clarity regarding antimicrobial selection for the management of M. *genitalium*, its routine screening in international backpackers is difficult to support given the low prevalence, uncertainties regarding contact tracing and difficulty with adequate follow up.

The prevalence of *C*. *trachomatis* in our study was comparable to a number of other studies in Australia, with one Australian review reporting a mean *C*. *trachomatis* prevalence of 4.6% [52]. This was consistent with previous population-level studies of international backpackers in Australia, with 4.3% reported in one recent study and 3.5% in another [30,32]. In two other Australian studies utilising population-level sampling of international backpackers, *C*. *trachomatis* rates were higher than reported in this study, at 11.9% in unsupervised screening and 7.7% in sexually active backpackers, both at hostels [31,35]. Higher rates of *C*. *trachomatis* infection in international backpackers have been reported, but these studies represented treatment-seeking groups attending sexual health clinics. Our study has been unable to replicate previously-reported associations between age, rates of partner change and inconsistent condom use to *C*. *trachomatis* positivity amongst backpackers. This may be a reflection on the small sample size and relatively few positive samples obtained, although our study is not the first to be unable to make any significant association between a variety of sociodemographic factors and sexual behaviours with *C*. *trachomatis* positivity in backpackers [32,34]. Inconsistent condom use, reporting 2 or ≥3 sexual partners during travel and reporting a known STI contact were risk factors for *C*. *trachomatis* infection in male backpackers in one study, however these may not differ from non-backpacker groups [33].

Prevalence of *N*. *gonorrhoeae* in our study was also equivalent to prevalence seen in other population-level studies screening international backpackers, with 0% reported in two studies, and 0.72% in another study of health-seeking backpackers accessing sexual health services; this is likely due to the fact that *N*. *gonorrhoeae* is predominately symptomatic and the sample group was mostly asymptomatic [30,31,34].

*M*. *genitalium* was associated with reporting STI symptoms, but not in *C*. *trachomatis* infection, in our sample. Infection with *C*. *trachomatis* was weakly associated with arriving in Australia with a sexual partner. This outcome is likely due to skewed data, as two of the participants testing positive for *C*. *trachomatis* were in a long-term monogamous relationship with each other. The number of sexual partners engaged with in Cairns was also associated with *C*. *trachomatis* infection, but not the number of sexual partners engaged with in any other location. Engaging in sex with multiple partners whilst in Cairns was reported by 54.5% of those testing positive for *C*. *trachomatis*. This may suggest that backpackers actively engage in regular sexual health checks, as rates of partner change are only significantly correlated with *C*. *trachomatis* positivity at the most recent travel destination, suggestive of recently obtained infection as opposed to asymptomatic carriage. However regular sexual health seeking behaviour has not been the case in previous reports [30].

Owing to the high frequency of recent antibiotic use, there exists the potential that the prevalence of STIs in this population may be greater than reported. This is especially relevant when considering multiple serovars of *C*. *trachomatis* remained susceptible (in varying degrees) to macrolides, tetracyclines, fluoroquinolones and β-lactam antibiotics in a recent *in vitro* study [53]. Having a past STI diagnosis was reported by 21.6% of participants, consistent with the notion that international travellers report a past diagnosis of STI more often than non-travelling population (15% vs 10%, respectively, p < 0.001), in one Australian study [34].

This study is limited in that the only specimens collected were FVU samples. Evidence suggests that for females, vaginal swab specimens are the most sensitive specimen for the detection of *M*. *genitalium* (despite the absence of an established gold standard method for detection of *M*. *genitalium*), followed by cervical swabs and then urine samples (κ = .923, κ = .843 and κ = .687 respectively), with another study reporting the sensitivity of 85.7% for vaginal swab samples, 74.3% for endocervical swabs, 61.4% for urine and 24.3% for rectal swabs [54,55]. This may have resulted in an under-representation of *M*. *genitalium*-positive females in our study. The overall prevalence of *M*. *genitalium* in this population may be higher if other anatomical sites were included. This study is also limited in that a number of other known STIs were not investigated. This was due in part to the financial and logistical constraints of collecting blood samples in a hostel environment and due to a number of other STIs existing beyond the scope of this project. An additional limitation of this study was the number of samples that were unable to be assessed owing to the presence of inhibitors of PCR in the urine. There exists the possibility that subjecting specimens to freeze-thaw cycles may alter the sensitivity of PCR testing. The impact of this limitation could have been decreased with the utilisation of DNA/RNA stabilising agents, allowing samples to be stored at ambient temperature [54]. The sensitivity of the detection of STIs is influenced by the time since last micturition and the participant’s ability to correctly collect a FVU sample, and in females factors such as degree of contamination of urine samples with cervical or vaginal secretions may increase the sensitivity of *M*. *genitalium* testing [54]. Further limitations include not assessing additional sexual risk behaviours, such as: alcohol or drug use, time since last STI check, types of sexual activity, or whether participants were utilising the services of sex workers. Participants were also not asked if they were engaging in sex with Australian citizens or other backpackers, which limits this study’s capacity to assess the risk of transmission of STIs and antibiotic resistance from international sources to Australians or vice versa. Another limitation of this study is that there was no formal follow up of STI-positive participants, and therefore we are unable to comment on the relationship between microbiological and clinical macrolide resistance. This study was also limited by the small sample size, and unexpectedly low number of participants testing positive for STIs.

*M*. *genitalium* and *C*. *trachomatis* were relatively common STIs in our cohort of non-treatment-seeking international backpackers in Cairns, Queensland Australia. International backpackers appear to be a relatively heterogeneous group, and research into this collective may not describe backpackers as a whole. The overall paucity of evidence supporting the categorisation of international backpackers as a high-risk group for STIs and the small volume of evidence suggesting no difference in (and in the case of *M*. *genitalium*, reduced) prevalence of STIs when compared to non-travelling groups, makes the justification for outreach sexual health clinics in hostels unfavourable. Concerns regarding the transmission of antibiotic-resistant strains of STIs are supported by a cumulative body of evidence that proposes macrolide resistance-associated mutations are becoming increasingly prevalent in *M*. *genitalium* [3,24,26,50]. The unease regarding the spread of antibiotic resistance is heightened when considering the behaviours of sexually active itinerant groups with local populations, and even more so when considering the potential for empirical therapies to induce macrolide resistance in otherwise sensitive *M*. *genitalium* strains [7,32,56–58]. Current guidelines regarding the treatment of NGU may be outdated regarding antibiotic resistance patterns, resulting in an environment where selection pressure for the development of resistance is intensified. Despite this evidence, the empirical management of NGU in Australia continues to utilise a 1 gram stat dose of azithromycin, with the intent being to treat chlamydial NGU, which is thought to cause more serious long-term sequelae [59]. However, evidence suggests the potential for significant morbidity associated with *M*. *genitalium*, especially in females, and has resulted in appeals within the literature for *M*. *genitalium* to be regarded with more significance [7,59,60]. Our research provides continued support for the implementation of now commercially available assays which simultaneously detect *M*. *genitalium* and macrolide resistance and regular screening of high risk groups, a risk category international backpackers may inhabit [59]. However, the data does not support a clear public health benefit from hostel-based sexual health outreach services or the regular screening of low to medium risk asymptomatic individuals for *M*. *genitalium*.

## Supporting information

S1 File Survey questions. (DOCX)
