# Supplementary material for: Mycoplasma genitalium in the Far North Queensland backpacker population: An observational study of prevalence and azithromycin resistance
Source: PLoS One. 2018 Aug 28;13(8):e0202428. doi: 10.1371/journal.pone.0202428 (PMC6112622; doi:10.1371/journal.pone.0202428)
Supplement: S1 File — (DOCX) [file pone.0202428.s001.docx]

#
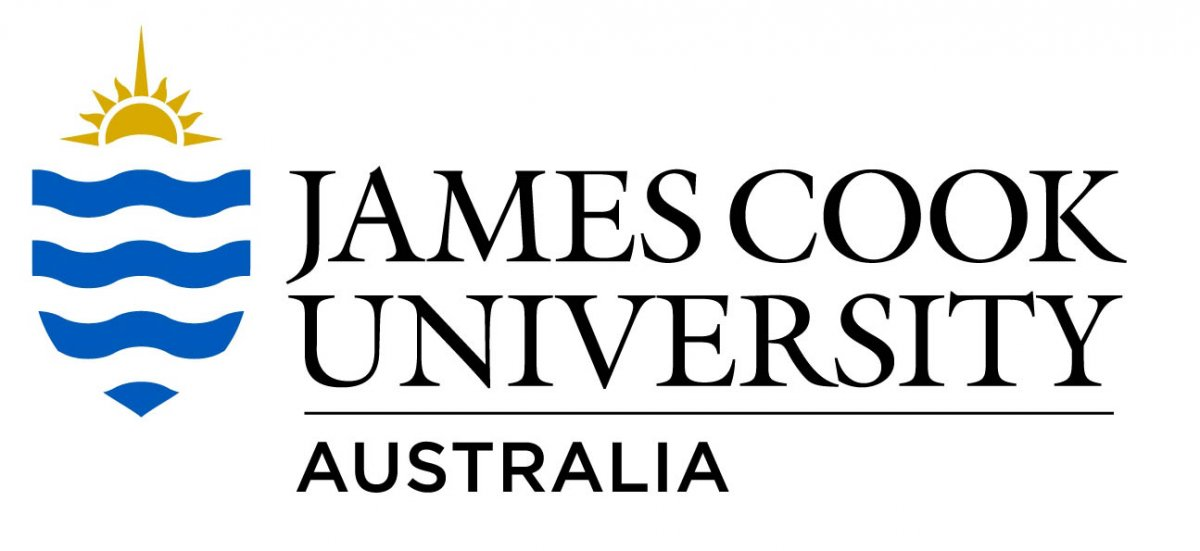
Sexually Transmissible Infections In Far North Queensland Backpackers Questionnaire

#### M. genitalium in the Far North Queensland backpacker population: prevalence & azithromycin resistance in relation to travel history & sexual behaviour

1. What is your age?
   1. 18 – 20 years
   2. 21 – 24 years
   3. 25 – 35 years
   4. > 35 years
2. What is your gender?
   1. Male
   2. Female
   3. Other
3. Since arriving in Australia, have you stayed at least one night in backpacker or hostel accommodation?
   1. Yes
   2. No
4. Did you arrive in Australia with a partner?
   1. Yes
   2. No
5. How long have you been in Australia?
   1. < 2 weeks
   2. 2 – 4 weeks
   3. 4 – 12 weeks
   4. > 12 weeks
6. How long have you been in Cairns?
   1. < 2 weeks
   2. 2 – 4 weeks
   3. 4 – 12 weeks
   4. > 12 weeks
7. What is your total expected stay in Australia?
   1. < 2 weeks
   2. 2 – 12 weeks
   3. 12 – 40 weeks
   4. > 40 weeks
8. What is your total expected stay in Cairns?
   1. < 2 weeks
   2. 2 – 12 weeks
   3. 12 – 40 weeks
   4. > 40 weeks
9. What is the approximate number of sexual partners you engaged with in the 12 months prior to leaving your country of origin?
   1. *Number*
10. What is the approximate number of sexual partners you have engaged with during your stay in Australia?
    1. *Number*
11. What is the approximate number of sexual partners you have engaged with during your stay in Cairns?
    1. *Number*
12. Of the sexual partners you engaged with in Australia, with how many did you not use a condom?
    1. *Number*
13. Did you visit any other countries prior to visiting Australia?
    1. Yes
    2. No
14. If yes to the last question, what country/countries were visited?
    1. *Name of country/countries*
15. If yes to the last question, how long did you stay in each country?
    1. *Number*
16. If yes to the last question, what is the approximate number of sexual partners you engaged with while in each country?
    1. *Number*
17. Of the sexual partners you engaged with overseas, with how many did you not use a condom?
    1. *Number*
18. Have you ever been diagnosed with, or treated for, a sexually transmissible infection in the past?
    1. Yes
    2. No
    3. Unsure
19. At the present time, do you have any symptoms such as pain when urinating, pain with sexual intercourse, discharge or other symptoms that make you believe you could have a sexually transmissible infection?
    1. Yes
    2. No
    3. Unsure
20. In the last three months have you been treated with antibiotics for any illness?
    1. Yes
    2. No
21. If yes to the above question, can you recall which antibiotic was prescribed to you?
    1. *Antibiotic*
22. I wish to receive a copy of the resulting publication/thesis via email at the completion of this project.
    1. Yes
    2. No
